# Association between Regulator of G Protein Signaling 9–2 and Body Weight

**DOI:** 10.1371/journal.pone.0027984

**Published:** 2011-11-23

**Authors:** Jeffrey L. Waugh, Jeremy Celver, Meenakshi Sharma, Robert L. Dufresne, Dimitra Terzi, S. Craig Risch, William G. Fairbrother, Rachael L. Neve, John P. Kane, Mary J. Malloy, Clive R. Pullinger, Harvest F. Gu, Christos Tsatsanis, Steven P. Hamilton, Stephen J. Gold, Venetia Zachariou, Abraham Kovoor

**Affiliations:** 1 Department of Psychiatry, University of Texas Southwestern Medical Center at Dallas, Dallas, Texas, United States of America; 2 Department of Biomedical and Pharmaceutical Sciences, University of Rhode Island, Kingston, Rhode Island, United States of America; 3 Kovogen LLC, Mystic, Connecticut, United States of America; 4 Department of Pharmacy Practice, University of Rhode Island, Kingston, Rhode Island, United States of America; 5 Department of Basic Sciences, Faculty of Medicine, University of Crete, Heraklion, Crete, Greece; 6 Department of Psychiatry, University of California San Francisco, San Francisco, California, United States of America; 7 Department of Molecular and Cell Biology and Biochemistry, Brown University, Providence, Rhode Island, United States of America; 8 Department of Brain and Cognitive Sciences, Massachusetts Institute of Technology, Cambridge, Massachusetts, United States of America; 9 Cardiovascular Research Institute, University of California San Francisco, San Francisco, California, United States of America; 10 Department of Medicine, University of California San Francisco, San Francisco, California, United States of America; 11 Department of Biochemistry and Biophysics, University of California San Francisco, San Francisco, California, United States of America; 12 Department of Physiological Nursing, University of California San Francisco, San Francisco, California, United States of America; 13 Department of Molecular Medicine and Surgery, Karolinska University Hospital, Karolinska Institutet, Stockholm, Sweden; Emory University, United States of America

## Abstract

Regulator of G protein signaling 9–2 (RGS9–2) is a protein that is highly enriched in the striatum, a brain region that mediates motivation, movement and reward responses. We identified a naturally occurring 5 nucleotide deletion polymorphism in the human *RGS9* gene and found that the mean body mass index (BMI) of individuals with the deletion was significantly higher than those without. A splicing reporter minigene assay demonstrated that the deletion had the potential to significantly decrease the levels of correctly spliced *RGS9* gene product. We measured the weights of rats after virally transduced overexpression of RGS9–2 or the structurally related RGS proteins, RGS7, or RGS11, in the nucleus accumbens (NAc) and observed a reduction in body weight after overexpression of RGS9–2 but not RGS7 or 11. Conversely, we found that the RGS9 knockout mice were heavier than their wild-type littermates and had significantly higher percentages of abdominal fat. The constituent adipocytes were found to have a mean cross-sectional area that was more than double that of corresponding cells from wild-type mice. However, food intake and locomotion were not significantly different between the two strains. These studies with humans, rats and mice implicate RGS9–2 as a factor in regulating body weight.

## Introduction

A large body of data indicates that brain circuits in the striatum that utilize opioid peptides and dopamine as neurotransmitters are important in i) the motivation to acquire food, ii) encoding food value and reward and iii) the orchestration of movements for acquiring food [1], [2].

Regulators of G-protein signaling (RGS) are a family of proteins that can accelerate GTP hydrolysis catalyzed by G protein coupled receptor (GPCR)-activated, Gα G protein subunits. Consequently, they accelerate the termination of GPCR signals [3]. In addition to the class-defining RGS domain which is responsible for the GTPase accelerating function (GAP) function, RGS proteins also contain additional regions that mediate intracellular interactions and non-canonical functions that are distinct from the canonical GAP function [4].

This study which investigates the role of the striatally enriched RGS protein, RGS9**–**2 [5], in regulating body weight was prompted by the following findings. First, RGS9–2 specifically modulates the GPCRs, D2-like dopamine receptors (D2R) [6], [7], [8], [9], and mu opioid receptors [10], [11], [12]. Second, D2R and mu opioid receptor signaling in the striatum regulates feeding behavior, body weight [1], [2], and reward responses [13], [14]. Third, several studies have shown that altered RGS9–2 levels modulate the reward responses to drugs that activate brain opioid and dopamine receptors [15].

RGS9–1 and RGS9–2 are the short and long splice variants, respectively, of the *RGS9* gene and the expression of each of these variants is highly tissue specific. RGS9–2 is expressed specifically in the brain and is highly enriched in striatum neurons while RGS9-1is thought to be expressed specifically in the retina [5], [16], [17]. RGS9–1 and 2 are members of the R7 RGS protein subfamily [18] whose members are defined by the presence of two N-terminal domains: i) a DEP (for dishevelled/EGL-10/pleckstrin homology) domain and ii) Gγ-like domain (GGL) that binds Gβ5, an outlying member of the G protein beta subunit family.

Here, we identify a polymorphism in the human RGS9 gene likely to alter functional levels of RGS9–2. Based on our observations that *RGS9* knockout mice were heavier than their wild-type littermates we asked if this human RGS9 gene polymorphism was associated with altered body mass index. We also tested the effects of virally-mediated overexpression of RGS9–2 in the rat nucleus accumbens (NAc) on body weight. The results from these experiments, involving humans, rats and mice, suggest that alterations in functional levels of RGS9–2 can affect body weight.

## Results

### Humans with a naturally occurring intronic deletion in the RGS9 gene have significantly higher body mass index (BMI)

We identified a naturally occurring deletion polymorphism (denoted here as Δ_TTTCT_) in intron 13 of the human *RGS9* gene. This human *RGS9* gene polymorphism has been reported previously as rs3215227 in the Single Nucleotide Polymorphism database, (dbSNP, http://www.ncbi.nlm.nih.gov/snp), and matched perfectly to a recently defined binding motif [19] for the ubiquitously expressed RNA binding protein, polypyrimidine tract binding protein (PTB). PTB was originally identified as a protein with an important role in RNA splicing [20], and is now known to function in a large number of diverse cellular processes including polyadenylation, mRNA stability and translation initiation.

Removal of introns from RNA transcripts and the splicing of flanking exons to produce the mature mRNA occur via the coordinated recognition of important sequence elements within the RNA transcript by an RNA-protein complex known as the spliceosome. Important sequence elements within the RNA transcript that direct the splicing of the transcript include the branch point, the 5′ splice site, the polypyrimidine tract, and the 3′ splice site. These sequence elements also serve as binding sites for additional factors such as PTB that modulate the splicing of specific transcripts [20].

The *RGS9* deletion polymorphism, Δ_TTTCT_, lies within a polypyrimidine tract, 72 nucleotides upstream of exon 14 and only 3 bases away from the branch point. The location of the Δ_TTTCT_ polymorphism and coincidence with a PTB binding motif suggested that the presence of the deletion could alter processing of *RGS9* transcripts and have important functional consequences: exons 13 and 14, that flank the Δ_TTTCT_ deletion, code for the conserved RGS domain that mediates GTPase accelerating protein (GAP) function [3], [17], [18]. Hence, incorrect splicing of these exons will result in protein products that lack a functional RGS domain.

Since, i) the Δ_TTTCT_ deletion polymorphism had the potential to reduce the functional levels of RGS9 protein and ii) our preliminary results indicated that *RGS9* knockout mice were heavier than their wild-type littermates, we hypothesized that humans with the Δ_TTTCT_ polymorphism were on average heavier than those who were negative for the polymorphism.

A preliminary screen indicated that the Δ_TTTCT_ deletion polymorphism was rare in Caucasians and African Americans, occurring at a frequency of less than 1%. As a result of the infrequency of the deletion in these ethnic groups we estimated that we would not be able identify enough Δ_TTTCT_ deletion positive Caucasians or African Americans to test for an association between the Δ_TTTCT_ polymorphism and body weight.

However, our preliminary screen suggested that Δ_TTTCT_ was more common in East Asians occurring at significantly higher frequencies. Therefore this ethnic group was studied to test for a relationship between the Δ_TTTCT_ deletion and body weight. We genotyped 491 individuals of Chinese, Filipino, Japanese, Korean, Pacific Islander, or Southeast Asian ancestry from whom blood samples had previously been obtained along with height, weight, age and sex data.

Forty nine individuals were homozygous for the Δ_TTTCT_ deletion (Δ_TTTCT_+/+), 197 individuals were heterozygous for the deletion (Δ_TTTCT_+/-), and 245 individuals tested negative for the deletion (Δ_TTTCT_-/-). The mean body mass index (BMI) was 25 (SD = 4.5) for the group homozygous for the deletion and 24.9 (SD = 4.4) for individuals heterozygous for the deletion. The mean BMI of the 245 individuals that tested negative for the deletion (Δ_TTTCT_-/-) was 24.2 (SD = 4.1) and was significantly different (t = 1.99, p<0.05), from the mean BMI, 24.9 (SD = 4.4), of the 246 individuals that tested positive for the deletion (Δ_TTTCT_+ve, i.e. individuals either homozygous, Δ_TTTCT_+/+, or heterozygous, Δ_TTTCT_+/-, for the deletion.

Table 1 outlines the ethnic sub-composition of the East Asian population sample, the frequency of the occurrence of the deletion in each sub-group and the mean BMIs of the Δ_TTTCT_+ve and Δ_TTTCT_-ve individuals in each group. The frequency of the incidence of the deletion was significantly different (χ^2^ = 16, p<0.005) between some of the different ethnic groups examined: those having Japanese and Korean ancestry had the lowest frequency of occurrence of Δ_TTTCT_.

**Table 1:** Table outlining the body mass index (BMI) associated with each ethnic group and with ΔTTTCT deletion positive (Δ+) and ΔTTTCT (**Δ**-) deletion negative groups.

	*BMI(Mean*±*SD) (No. of Subjects)*		
Ethnicity	*Δ* *+*	*Δ* *-*	*Δ+ & Δ-*
**Chinese** (45.0%)^a^	24.4±4.6 *(113)* b	23.2±3.5 *(108)* b	^†^23.8±4.1 *(221)* b
**Japanese** (15.9%)^a^	25.1±5.6 *(31)* b	24.4±4.2 *(47)* b	24.6±4.8 *(78)* b
**Korean** (4.7%)^a^	24.3±3.6 *(4)* b	23.8±4.1 *(19)* b	^§^23.9±4.0 *(23)* b
**Pacific Islander** (21.0%)^a^	25.9±3.9 *(61)* b	25.7±4.2 *(42)* b	25.8±4.0 *(103* b
**Southeast Asian** (13.4%)^a^	25.1±3.3 *(37)* b	25.3±5.0 *(29)* b	25.2±4.1 *(66)* b
**All East Asians** (100%)^a^	# ***24.9***±4.4 *(246)* b	***24.2***±4.1 *(245)* b	24.6±4.2 *(491)* b

Table Key:

***Δ+***: ΔTTTCT deletion positive; ***Δ-***: ΔTTTCT deletion negative

a Percentages in parenthesis in the first column indicate percent fraction of the study population that belong to each ethnic group.

b Numbers in parenthesis in columns 2–4 indicate the number of subjects associated with each cell.

†, § Ethnic groups have significantly different BMI's (ANOVA, F = 4.71, P<0.005); follow-up Fisher's Least Significant Difference test (P<0.05) test shows **^†^**Chinese have lower BMI than Southeast Asians or Pacific Islanders and **^§^**Koreans having a lower BMI than Pacific Islanders.

# Student independent t-test shows significant difference (p<0.05) between ΔTTTCT deletion positive (Δ+) and ΔTTTCT deletion negative (Δ-) individuals.

Analysis of variance of the relationship of ethnicity among the East Asian subjects on body mass index shows significant differences between those identifying themselves as Chinese, Japanese, Korean, Pacific Islander, or Southeast Asian. The different East Asian ethnic groups have significantly different BMI's (ANOVA, P<0.005); follow-up Fisher's Least Significant Difference test (P<0.05) test shows that Chinese have lower BMI than Southeast Asians or Pacific Islanders and Koreans have a lower BMI than Pacific Islanders. However, we found that within each ethnic group, the differences in the BMI between deletion-positive and deletion-negative individuals were not significant.

Men had a significantly higher (t = 6.56, p<0.001) BMI than women (25.8±3.6 versus 23.4±4.5, Mean±SD, Table 2), but, as would be expected by the chromosome 17 location of the RGS9 gene, there was no association (χ^2^ = 0.74) between sex and presence of the deletion and the deletion is similarly distributed in men and women. Therefore, unequal gender distribution between the deletion positive and negative groups does not account for the difference in BMI between the two groups. Finally, there was no relationship between BMI and age in our study population.

**Table 2:** Table outlining the mean body mass index (BMI) associated with males and females and with ΔTTTCT deletion positive (Δ+) and ΔTTTCT (Δ-) deletion negative male and female subjects.

	BMI (Mean±SD) (*No. of Subjects*)		
*SEX*	*Δ+*	*Δ-*	*Δ+ & Δ-*
**Females** (51.7%)^a^	23.9±4.8 (122)^b^	22.9±4.0 (132)^b^	23.4±4.5 (254)^b^
**Males** (48.3%)^a^	26±3.7 (124)^b^	25.6±3.6 (113)^b^	†25.8±3.6 (237)^b^
**All Subjects**	# ***24.9***±4.4 (246)^b^	***24.2***±4.1 (245)^b^	24.6±4.2 (491)^b^

Table Key:

***Δ+***: ΔTTTCT deletion positive; ***Δ-***: ΔTTTCT deletion negative

a Percentages in parenthesis in the first column indicate percent fraction of the study population that is either female or male.

b Numbers in parenthesis in columns 2–4 indicate the number of subjects associated with each cell.

† Men had a significantly higher (Student independent t-test, t = 6.56, p<0.001) BMI than women.

# Student independent t-test shows significant difference (p<0.05) between ΔTTTCT deletion positive (Δ+) and ΔTTTCT deletion negative (Δ-) individuals.

A more detailed description of the East Asian study subjects are provided in the Supporting Information Tables S1 and S2.

### Effects of the Δ_TTTCT_ deletion polymorphism on splicing of minigene construct

Since the Δ_TTTCT_ deletion was close to the branch point for RNA splicing and corresponded to a binding motif for PTB, we attempted to determine if the deletion could alter splicing of the *RGS9* gene transcript.

Analysis of RNA from the tissue of individuals with the Δ_TTTCT_ polymorphism would be the ideal method of assessing the effect of this deletion on splicing. However, RNA extracted from tissues of Δ_TTTCT_+ve individuals was not available to us. In addition, the splicing of the RGS9 transcript is highly tissue specific. For example, in humans, the shorter alternatively spliced form, RGS9-1 is highly expressed in the retina but not detectable in the brain, while RGS9-2 is largely expressed in the brain [17] and not detectable in the retina. Therefore it is not certain that the effect of Δ_TTTCT_ on splicing of RGS9 RNA in an accessible tissue such as blood would be reflective of the splicing in the tissues responsible for the increased BMI phenotype. Furthermore, the *RGS9* gene transcript is very large (>170 kilobases) [17] and splicing alterations observed *in vivo* for such large transcripts cannot be definitively linked to a particular gene variation without sequencing the entire gene sequence for all the individuals in the study sample. In practice, such extensive sequencing was not possible and therefore we would not definitively exclude the possibility that the altered splicing arose, instead, from alternative undiscovered variations in non-sequenced sections of the gene.

**Figure 1:**
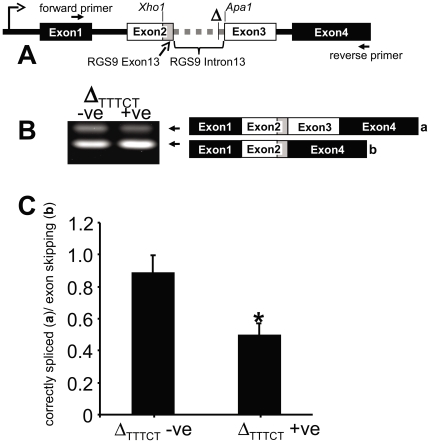
Effect of RGS9 gene elements containing the Δ_TTTCT_ deletion on the splicing of a minigene reporter construct. (**A**) Schematic representation of the minigene reporter system used to assess the effect of the intron 13 TTTCT deletion (represented as Δ) on RGS9 gene splicing. A section of the RGS9 gene spanning the 3′ end of exon 13 (gray shaded box) and intron 13 (gray dashed line between Exon 2 and 3) was inserted at the indicated restriction enzyme sites. Note that the 3′ end of the RGS9 gene exon 13 (gray shaded box) forms the 3′ end of Exon 2 of the new minigene construct. In subsequent panels “Δ_TTTCT_+ve” refers to the minigene construct made by inserting the RGS9 gene fragment with the TTTCT deletion while “Δ_TTTCT_-ve” refers to a minigene construct that is identical to Δ_TTTCT_ except that it does not have the TTTCT deletion. (**B**) The Δ_TTTCT_-ve and Δ_TTTCT_+ve RGS9 gene fragment-containing minigenes were transfected separately into HEK293 cells. The minigene transcripts were visualized by reverse transcription, PCR amplification followed by agarose gel electrophoresis. The gel image (left) is a representative image of DNA bands produced from the separate minigene transcripts that are depicted schematically on the right (labeled “**a**” and “**b**”, respectively). The binding sites of the PCR primers used to amplify “**a”** and “**b”** are indicated by the forward and reverse arrows in **A**. We sequenced bands “**a**” and “**b**” to determine that they are derived, respectively, from the minigene products shown schematically to the right of the gel image. (**C**) Densitometric quantification of the relative intensities of bands “**a**” and “**b**”, (*p<0.01, t test).

To circumvent such constraints splicing reporter minigene assays are often used to assess the impact of allelic variants on splicing [21], [22]. These assays examine the splicing of a minigene reporter constructed using the portion of the gene containing the variation and allow for a more definitive linkage of the gene variation to splicing alterations. As described in Methods and Materials and depicted schematically in Fig. 1A, an *RGS9* gene fragment containing exon 13 and intron 13 was utilized to construct a splicing reporter minigene which was transfected into HEK293 cells. The resulting gene transcripts were analyzed by reverse transcriptase PCR (RT-PCR) and gene sequencing. Incorporation of Δ_TTTCT_ deletion into the *RGS9* minigene fragment increased the frequency of exon skipping during splicing of the minigene transcript and decreased the fraction of the correctly spliced transcript (i.e. transcript containing all 4 minigene exons, Fig. 1B & C).

### Deletion of the RGS9 gene produces heavier mice

**Figure 2:**
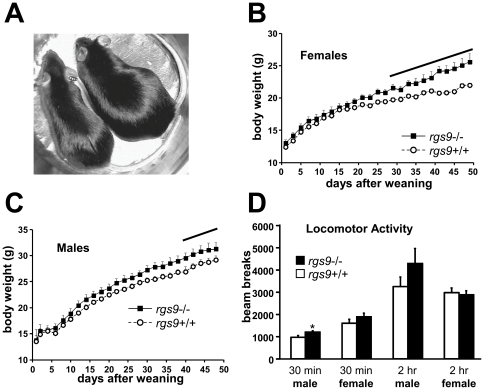
Body weight and locomotor activity comparisons of RGS9 wild-type (*rgs9*+/+) and RGS9 knockout mice (*rgs9*-/-). (**A**) 12 wk old male *rgs9-/-* (on right) with wild-type, *rgs9+/+* littermate at left. (**B**) Comparison of weight of female *rgs9-/-* and *rgs9+/+* littermates from weaning through day 50 post-weaning (number of mice at day 50, *rgs9+/+* 9, *rgs9-/-* 6). (**C**) Comparison as in **B** with male mice (number of mice, *rgs9+/+* 11, *rgs9-/-* 8). Points represent mean±SEM. Solid line, p<0.005, repeated measures ANOVA. (**D**) Comparison of ambulatory locomotor activity of 8 week old male and female *rgs9-/-* (black bars) and *rgs9+/+* (open bars) mice, after placement of mice in activity measurement chambers over a 2 hr period. Bars represent mean±SEM number of beam breaks per mouse after 30 min and after 2 hr (number of male mice, *rgs9+/+* 8, *rgs9-/-* 8, number of female mice, *rgs9+/+* 8, *rgs9-/-* 8, *p<0.02, unpaired t test male mice, *rgs9+/+* compared to *rgs9-/-*).

The weight disparity between the *RGS9* knockout (*rgs9*-/-) and wild-type (*rgs9+/+*) littermates is readily apparent by visual inspection – *RGS9* knockout mice are heavier (Fig. 2A).

To quantify the development of this phenotype, we recorded the weight of wild-type and homozygous *RGS9* knockout (*rgs9*-/-) mice born from heterozygote breeders that were given *ad libitum* access to water and 4% fat rodent chow. Mice were weighed daily from weaning until post-weaning day 55 (postnatal days 21 through 76).

The weights of both male and female *rgs9*-/- diverged from their wild-type littermates, at approximately 19 g for females and 21 g for males, and this disparity increased through day 50 post-weaning (Fig. 2B and C). Though we did not systematically measure body weight past day 55, observation of older animals, indicated that the increased weight of RGS9 knockout animals persisted at least through 12 months of age.

A measurement of the food consumed indicated that the altered weight of the knockout mice was not due to increased food intake. The amounts of food consumed by the *rgs9*-/- and *rgs9*+/+ were similar: 3.22±0.29 and 3.07±0.29 g/day (Mean±SEM) for the wild-type mice and the *RGS9* knockout mice, respectively.

The locomotor activity of the wild-type and *RGS9* knockout male and female mice measured over a 2 hr time period was similar (Fig. 2D). At an earlier time point (30 min) the male *rgs9*-/- mice demonstrated a small but significantly increased ambulatory activity when compared to their wild-type counterparts. This small increase in initial activity could represent an increased anxiety phenotype for these mice, but cannot easily explain their increased weight.

### Enlarged fat pads and adipocytes in RGS9 knockout mice and undetectable levels of RGS9 protein expression in visceral fat depots of wild-type mice

**Figure 3:**
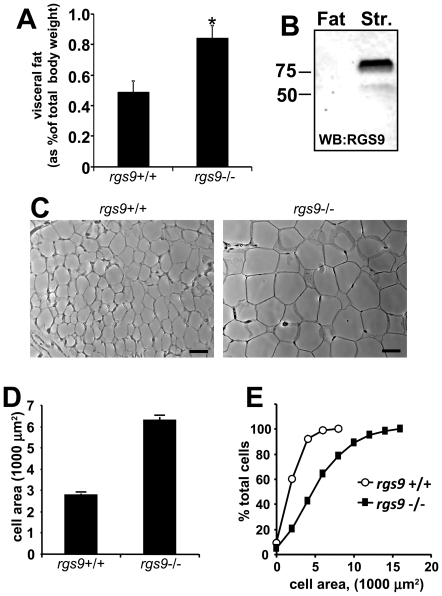
Comparison of visceral fat and mesenteric adipocytes from RGS9 wild-type (*rgs9*+/+) and RGS9 knockout mice (*rgs*9-/-). (A) Combined weight of epididymal and mesenteric fat depots expressed as a percentage of body weight. (number of mice, *rgs9+/+* 5, *rgs9-/-* 6,*p<0.02, t test). (B) Western blot (WB) of visceral fat (Fat) and striatal (Str.) tissue from an 8 week old female wild-type mouse probed with an antibody directed against RGS9. Numbers to the left of the blot indicate position of the 75 kDa and 50 kDa protein molecular weight markers. The blot is representative of experiments performed with 2 female and 2 male mice. (C) representative images of epididymal adipocytes from *rgs9+/+* (*left panel*) and *rgs9-/-* (*right panel*) mice. Scale bar represents 50 µm. (D) Apparent area of adipocytes observed in hematoxylin and eosin stained epididymal fat sections. n, *rgs9+/+* 463 cells, *rgs9-/-* 497 cells pooled from sections from two separate animals each, (*p<0.01, t test). (E) Cumulative frequency distribution of apparent epididymal adipocyte cross-sectional area. Adipocyte cross-sectional area is plotted on the x-axis, and y-axis indicates the percent of total cells with cross-sectional area less than the value indicated on the x-axis.

We dissected visceral white adipose tissue from mesenteric, epididymal and perirenal depots from age-matched wild-type and *RGS9* knockout male mice between 2 and 4 months old and found significantly larger amounts of visceral adipose tissue in the *RGS9* knockout mice (Fig. 3A).

The epididymal adipose tissue from 8 week old male or female wild-type mice was Western blotted and probed with a validated antibody previously shown to recognize both the long and short RGS9 isoforms [23]. While RGS9-2 protein expression was clearly detected in striatal tissue, we were unable to detect any RGS9 protein signal in the visceral fat from the same mouse using this approach (Fig. 3B). We also further validated the antibody by confirming that no protein signal was detected when Western blots of striatal tissue from the *rgs9*-/- mice were probed with the antibody (data not shown).

An analysis of the epididymal (Fig. 3C, D and E) or mesenteric depots showed that the adipocytes from *RGS9* knockout animals were significantly enlarged when compared to the wild-type animals (Fig. 3D and E).

### RGS9-2 over-expression in rat nucleus accumbens (NAc) leads to weight loss

Following our observations of the increased weight of *RGS9* knockout mice, we tested whether overexpression of RGS9-2 in the NAc could produce weight loss. We used a previously validated Herpes Simplex Virus (HSV) delivery system to drive overexpression of RGS9-2 in the nucleus accumbens (NAc) shell of male rats [7], [24] while quantifying their body weight dynamics. The effects of RGS protein overexpression on body weight were compared with the effects of control LacZ expression.

**Figure 4:**
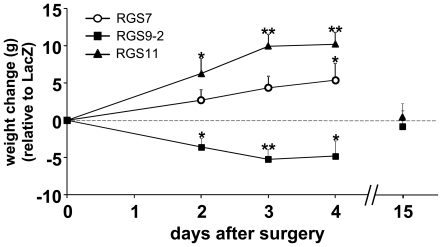
Body-weight changes in rats following Herpes simplex virus (HSV)-mediated over-expression of RGS proteins in the nucleus accumbens shell. Graphs depict the difference in body weight following intracranial injection of HSV constructs encoding RGS7 (*open circles*), RGS9-2 (*filled squares*) and RGS11 (*closed triangles*) into the nucleus accumbens (day 0), relative to the weight of control group HSV-LacZ-injected rats. The weights at day 0 for each of the paired comparisons were as follows: LacZ, 281.7±4.4 (n = 18); RGS7, 283.3±2.7 (n = 20); LacZ, 289.7±3.6 (n = 51); RGS9-2, 290.2±4.26 (n = 48); LacZ, 287.5±3.2 (n = 19); RGS11 292.2±3.4 (n = 22). Not all the animals were followed until day 15, hence “n”s at day 15 were as follows: LacZ, 22; RGS9-2, 14; RGS11, 11. *p<0.05, **p<0.01, repeated measures ANOVA followed by Fisher's least squares test.

Rats injected with the HSV RGS9-2 construct exhibited enhanced weight-loss relative to the LacZ controls. The loss in weight of the HSV RGS9-2 injected rats relative LacZ was greatest on day 3 after surgery (Fig. 4) and averaged 5.17 g (1.7% of initial body mass).

It has been shown previously that HSV-transduced RGS9-2 protein expression in the rat NAc peaks on day 3–4 post-injection and rapidly declines thereafter [7]. The relative weight loss experienced by the HSV-RGS9-2 injected rats was similarly transient: on post-surgical day 5, the body weights of the RGS9-2 expressing animals were no longer significantly different from the LacZ overexpressing controls. By day 15 following viral injection, group weights were indistinguishable (Fig. 4).

In contrast, overexpression of the structurally similar proteins, RGS7 and RGS11, in rat NAc shell led to weight gain relative to LacZ overexpression (Fig. 4). For example, on day 3 post-surgery, RGS11 overexpressing rats weighed on average 10.8 g (3.6% of total body mass) more than the LacZ controls.

## Discussion

In this manuscript we provide data from human, mouse and rat studies that suggest that RGS9-2, a brain specific RGS family member, can regulate body weight and adiposity. First we link an intronic deletion in the RGS9 gene to increased body mass index in humans (BMI). We provide mechanistic insights into this association by showing i) that the intronic deletion is coincident with a binding motif for the polypyrimidine tract binding protein (PTB), a protein involved in regulating the processing of RNA and ii) that the intronic variation can alter the splicing of the RGS9 gene product. Further confirmation for a role of RGS9 in regulating body weight is provided by observations showing that RGS9 knockout mice are heavier than their wild-type littermates, have increased adiposity and adipocytes with approximately doubled cross-sectional area. Finally we identify a possible site for the action of RGS9-2 in regulating body-weight by showing that overexpression of RGS9-2 in the nucleus accumbens of rats can decrease body-weight.

A major finding of our study is that an intronic deletion polymorphism in the RGS9 gene (Δ_TTTCT_) is associated with significantly increased body mass index (BMI) in humans. The increase in mean BMI (24.9 versus 24.2) represents an increase in weight from 68.2 kg (150 lb) to 70 kg (154 lb), or a 2.7% increase in weight, for an individual 168 cm (5 ft 6 inch) tall. Thus, the influence of the Δ_TTTCT_ deletion polymorphism on body weight is quite mild: a World Health Organization panel has suggested that the obesity cut-off definition for Asians should be lowered to a BMI of 25 [25], compared to 35 for Caucasians, but the mean BMI of the Δ_TTTCT_ deletion positive East Asian individuals in our study is less than even this lowered BMI value.

An examination of Table 1 shows that the increase in mean BMI of the Δ_TTTCT_ deletion positive subjects relative to the deletion negative individuals is greatest among the Chinese, is very small in Pacific Islanders and is reversed in Southeast Asians. However, the above differences and the differences in BMI between deletion positive and negative individuals within each ethnic sub-group were not statistically significant. Nevertheless, it is interesting to note that since Pacific Islanders (Polynesians) are descendants of voyagers from Southeast Asia—either Taiwanese aborigines or former inhabitants of New Guinea and the surrounding islands [26], [27], they are more closely related genetically to Southeast Asians than to Chinese, Japanese or Koreans. Hence, it is possible that within these two former genetically similar ethnic groups, the Δ_TTTCT_ deletion is not correlated to BMI, or the effect of the deletion is masked by other factors. Future studies with larger samples sizes will be required to determine whether the effect of Δ_TTTCT_ deletion on BMI is restricted to a subset of ethnic groups.

The association between the Δ_TTTCT_ intronic deletion polymorphism in the RGS9 gene and increased BMI was studied in the East Asian population because the deletion was rare in other groups. However, these results do not imply that RGS9-2 contributes to body weight set-points only in this ethnic group. Other RGS9-related variations, including yet unidentified variations in the RGS9 gene and regulatory regions, may contribute to variations in cellular levels of RGS9-2 protein and thus modify body-weight in individuals regardless of ethnicity.

It is also interesting to note that the mean weights of the female wild-type and knockout mice diverged significantly from each other at an earlier time point than for the male mice (day 30 after weaning for the females versus day 40 after weaning for the males). In addition, close to the end of the experiment, on day 50, the mean percent increase in weight of the female knockout mice compared to the female wild-type mice was 16.5% while the mean percent increase for the male knockout mice relative to the wild-type mice was 6.8%, but this difference was not statistically significant. Similarly, the difference in mean BMI of the Δ_TTTCT_ deletion positive relative to the deletion negative East Asian individuals was greater in females than in males (Table 2) and the difference between the genotypes was marginally significant in females but not in males (p = 0.07 for females versus p = 0.45 for males, t test). Future studies with larger samples sizes will be required to determine whether the effect of *RGS9* gene variations on weight is more prominent in females.

The location of the Δ_TTTCT_ deletion polymorphism and the identity to a binding motif for the polypyrimidine tract binding protein (PTB) suggested that this naturally occurring allele could affect the splicing of RGS9 mRNA. Indeed, our data shows that the deletion can alter splicing and produces a substantial reduction in correctly-spliced RGS9 gene transcript (Fig. 1). The Δ_TTTCT_ polymorphism lies within intron 13. The exons that flank intron 13 code Exons 13 and 14 which encode the RGS domain [17]. The RGS domain is conserved among all the RGS family members and mediates the canonical Gα GTPase accelerating (GAP) function [3]. Thus, altered splicing due to the Δ_TTTCT_ deletion polymorphism, such as exon skipping or intron retention, will result in a protein product that is truncated at this region and therefore lacks the important class-defining GAP function. These results, in turn, suggest that individuals carrying the deletion polymorphism have a higher BMI due to a reduction in the functional levels of brain RGS9-2.

Support for the suggestion that RGS9-2 is important in regulating body-weight is provided by the finding that mice with the *RGS9* gene deletion have elevated body weight (Fig. 2) and that conversely overexpressing RGS9-2 in the rat nucleus accumbens (NAc), via herpes simplex virus (HSV)-mediated gene transduction, lowers body weight relative to control animals (Fig. 4). RGS9-2 is expressed in the vast majority of striatal medium spiny neurons [7], which comprise 90–95% of the neurons in the striatum [28]. Hence, the percentage of infected non-target cells that do not normally express RGS9-2 is small and consequently, this technique has been successfully utilized to define physiological and pathophysiological functions for RGS9-2 using both rodent and primate models [7], [24].

Indeed, the results presented above mirror those from previous studies, where HSV-mediated RGS9-2 over-expression in the rat striatum has been shown to produce functional responses that are opposite to those exhibited by the *RGS9* knockout mice. For example, *RGS9* knockout mice exhibit increased cocaine-induced locomotion, while HSV-mediated overexpression of RGS9-2 in rats dampens cocaine-induced locomotion [7]. In addition, *RGS9* knockout mice show accelerated development of drug-induced dyskinesia [6], while HSV-mediated overexpression of RGS9-2 in the striatum of rats and monkeys diminished intensity of drug-induced dyskinesia [24].

The specificity of RGS9-2 overexpression effects are highlighted by the parallel experiments with RGS7 and RGS11 [18], closely related members of the R7 RGS protein family. Though RGS11 is thought to be specifically expressed in retinal bipolar neuron, the two proteins have been shown *in vitro* to act as GTPase accelerating proteins for the same G proteins [18], [29], [30]. The opposite effects of RGS9-2 and RGS7 and RGS11 overexpression on body weight (Fig. 4) suggest that the effects cannot be attributed solely to the GAP function which is common to the three proteins. While we do not yet understand the mechanism for the opposing action of these proteins on body weight, it is interesting to note that R7 RGS proteins, such as RGS7, RGS9 and RGS11, can compete for their obligate binding partners (Gβ5 and R9AP or R7BP). In fact, knock-out of these binding partners leads to marked reduction in all R7 RGS proteins [31], [32], [33]. Therefore, one hypothesis explaining the relative increase in weight produced by RGS7 or RGS11 overexpression, is that RGS7 and RGS11 competes with RGS9-2 for the available Gβ5 and R7BP, destabilizing native RGS9-2 protein and producing an effect on weight similar to that seen in *RGS9* knock-out mice. Results similar to those we report have been observed at the cellular level: previous studies have reported that, despite their structural similarity, RGS7 and RGS11 produce opposite effects to those produced by RGS9 on cellular signaling pathways [34], [35].

While there is one report of RGS9 transcript expression in blood lymphocytes [36], all other studies examining the tissue distribution and function of RGS9 gene products have reported only on the expression of RGS9 in the brain and the retina [5], [17], [37], [38]. Thus, the absence of reports of RGS9 expression in peripheral tissue, interpreted in conjunction with, i) our observations with rats demonstrating that RGS9-2 overexpression in the NAc lowers body weight compared to control animals (Fig. 4) and ii) undetectable levels of RGS9 protein expression in fat tissue (Fig. 3B), suggest that the RGS9 acts to regulate body-weight and adiposity via expression in the brain.

RGS9-2 exhibits extremely dense expression in rodent striatum, and pleasure, desire and reward circuits operating in the striatum are thought to be important in obesity and eating disorders [39]. However, the actions of RGS9-2 in regulating body-weight and adiposity are likely to involve alternative striatal connections, since we report that the food intake of the heavier *RGS9* knockout mice was similar to that of wild-type mice.

The dopamine-sensitive NAc reward centers are densely interconnected with the hypothalamus, a brain region that is critical for regulating energy expenditure. Hypothalamic neurons, including orexin [40], [41] and melanin concentrating hormone neurons [42], make connections with the NAc, and the NAc can influence hypothalamic functions [43]. Thus, RGS9-2 via expression in the NAc could modulate the activity of hypothalamic centers that control energy expenditure and future experiments will examine the total and resting oxygen consumption in the *RGS9* knockout mice. Interestingly, in addition to extremely dense expression in striatum, RGS9-2 is also localized to medial hypothalamus [38]. However, it is clear from our experiments that modulating RGS9-2 levels specifically in the NAc can alter body weight (Fig. 4).

Human genetic studies have linked variations in two other R7 RGS proteins, RGS6 [44] and RGS7 [44] to obesity. The above human genetic studies provide a precedent for R7 RGS family proteins regulating body-weight through their expression in the brain since these proteins have been reliably detected only in the brain or in excitable tissue such as retina and heart [37], [45], [46].

More recently, it has been reported that mice with a targeted deletion of one copy of the G protein Gβ5 subunit gene are heavier and have increased adiposity when compared to their wild-type counterparts, even though their food intake was not different and their locomotor activity levels were enhanced [47]. Similarly, we found that the food intake of the heavier RGS9 knockout mice was not different from their wild-type littermates, and also observed a small but significant increase in initial activity of the male *RGS9* knockout mice when placed in the movement measurement cages (Fig. 2D). Gβ5 protects R7 RGS family proteins from proteolysis and expression of R7 RGS family proteins, including RGS9, is eliminated or hugely reduced in the absence of Gβ5 [18], [32]. Therefore our results raise the possibility that the increased adiposity and weight phenotype of heterozygous Gβ5 knockout mice is produced as a result of a Gβ5 knockdown-mediated reduction in brain RGS9-2 protein levels.

Human and mouse studies have also implicated RGS2, 4 and 5, in the regulation of body weight and obesity [48], [49], [50], [51]. However these RGS proteins and RGS9 likely regulate body weight through different mechanisms: RGS2, 4 and 5 are members of a different R4 subfamily of RGS proteins, have a different molecular architecture and strikingly different cellular and tissue expression patterns compared to RGS9 [18], [37], [52]. It has been reported that knock-in mice homozygous for a mutant Gα_i2_ G protein subunit that does not bind RGS protein are resistance to diet-induced obesity [53]. However, the molecular mechanism underlying the body-weight phenotype of these mice is also likely to be different from that in the *RGS9* knockout mice: Gαi2, is expressed ubiquitously in peripheral tissues and in many brain regions and does not have the restricted expression pattern of the *RGS9* gene products [53].

Previously, altered RGS9-2 levels have been shown to be involved in drug-addiction and the reward response to psychostimulants [15] and our results are significant because we implicate the same molecule in the control of body weight and adiposity.

## Materials and Methods

### Human genotyping

The human genotyping study was approved by the Human Research Protection Program Committee on Human Research of the University of California, San Francisco. The study application was titled, “Discovery of Gene Mutations and Polymorphisms that Contribute to the Risk of Cardiovascular and Metabolic Disease and Macular Degeneration”. The Institutional Review Board number was 10–00207 and the permit number was 016323. Written informed consent was obtained from all subjects.

DNA samples were analyzed for the presence of the Δ_TTTCT_ polymorphism from participants of East Asian ancestry, in the University of California, San Francisco, Genomic Resource in Arteriosclerosis (GRA) collection, which has been previously described [54], [55] and contains associated height, weight, sex and age data. The body mass index for each subject was calculated using the formula: *mass (kg) ÷ (height (m))* ^2^ [25].

The primer sequences used for detecting the Δ_TTTCT_ deletion polymorphism were, forward, 5′-GTGCAATAGCTTGTTCTGCG and reverse, 5′-TGGCAAGTACAGTGAACTGATG (chr17:63,197,962–63,198,341). Forward primers were fluorescently labeled with 6-FAM (Life Technologies-Invitrogen-Applied Biosystems, Carlsbad, CA). PCR was carried out using Platinum Taq polymerase (Invitrogen). The PCR reaction was performed on 10 to 25 ng of genomic DNA with 2.0 mM MgCl_2_, 0.25 units Taq, 200 µM dNTPs, 0.02 µM of each primer and PCR buffer supplied with the Taq enzyme. An ABI 3730 (Applied Biosystems) DNA analyzer was utilized for the initial Sanger sequencing visualization and fragment separation. The genotyping results were determined by visualization using STRand [56] while Sequencher (Gene Codes Corporation, Ann Arbor, MI) was used to analyze the sequencing results.

The statistical analysis of the human data was performed using IBM SPSS Statistics (IBM, Armonk, NY) and Systat 12 (Systat Software, Chicago, IL) software.

### Splicing reporter minigene assay

The reporter system used to screen for the possible effect on splicing of the Δ_TTTCT_ deletion polymorphism was derived from a plasmid vector containing a 3 exon minigene under the control of the human cytomegalovirus promoter and has previously been described by Wang and colleagues [57]. The first and last exons of the original 3 exon minigene consist of sequences that encode for portions of the enhanced green fluorescent protein (eGFP). These two exons are separated by a small constitutively spliced exon, of the Chinese hamster dihydrofolate reductase (DHFR) gene, together with its flanking introns. The DHFR exon also contains two restriction cloning sites, Xho1 and Apa1, into which we inserted a synthesized (Epoch Life Sciences, Missouri City, TX) fragment of the human *RGS9* gene [17]. A schematic of the minigene construct is shown in Fig. 1A. The inserted RGS9 gene fragment consisted of the final 57 base pairs of exon 13 (Fig. 1A, grey box,) followed by a fusion of the first 100 and the last 243 base pairs in the adjacent intron 13 (Fig. 1A, dashed grey bar). The insertion of the regions from intron 13 of the RGS9 gene effectively splits the central DHFR exon of the original minigene into 2 separate exons thus creating a new minigene construct with 4 exons (Fig. 1A). To investigate the putative effect of the Δ_TTTCT_ deletion on splicing, a second construct, identical to the one described above, was made except that the inserted RGS9 gene fragment was synthesized with Δ_TTTCT_ deletion (represented as Δ in Fig. 1A) at the appropriate intron 13 site. To increase the likelihood of detecting splicing effects of a polymorphism located outside the canonical splicing sites, we weakened the canonical splicing machinery near the 3′ splicing site (C→A, at position -3) and near the 5′ splicing site (G→T, last position in the exon). A similar strategy of weakening splicing signals or adding negative elements to impair the recognition of a test exon has been commonly used to test the effects of silencer or enhancer elements in other studies [21].

Human embryonic kidney cells (HEK293, American Type Culture Collection, Manassas, VA) were separately transfected with equal amounts of the ΔTTTCT deletion positive (+ve) or the ΔTTTCT deletion negative (-ve) minigene constructs described above. The transient transfections were performed using LTX transfection reagent (according to manufacturer's instructions Invitrogen, Carlsbad, CA).

After 48 hr the cells were harvested and total cellular RNA was isolated using TRIzol reagent (according to manufacturer's instructions, Invitrogen). Next, the RNA was reverse transcribed (reverse transcriptase enzyme, Sigma Aldrich) at 37 °C for 50 min in a reaction that was primed with random hexamer nucleotides and contained dNTP mixture and RNase inhibitor (Ambion) and buffer provided by the enzyme supplier.

The resulting cDNA was amplified in a PCR reactions (25 cycles) using Taq DNA polymerase (Promega, Madison, WI) and forward (5′ AAATGGAATCCATCCCGG 3′) and reverse (5′ AAACCCTAGAATCCTCATC 3′) primers flanking the first and last exon of the minigene such that splicing alterations resulting from exon skipping or intron retention would alter the size of the PCR fragment amplified. The identity of the alternatively spliced gene fragments were confirmed by sequencing.

### Rodent studies

Rodent studies were carried out in strict accordance with the recommendations in the Guide for the Care and Use of Laboratory Animals of the National Institutes of Health. All experimental protocols using rats and mice were approved by the Institutional Animal Care and Use Committee of the University of Texas Southwestern Medical Center at Dallas or the University of Rhode Island with protocol numbers, 0930-04-02-1 and 242945-1, respectively.

### Mouse Weight Measurements

Genotyping of the *rgs9-/-* mice was described previously [58]. Mice were backcrossed to C57BL/6J mice (Stock Number: 000664, Jackson Laboratory, Bar Harbor, ME) for four generations. An *rgs9+/-* X *rgs9+/-* breeding strategy was used to generate wild-type (*rgs9+/+*), heterozygous (*rgs9+/-*) and RGS9 knockout (*rgs9-/-*) littermates. At weaning, mice were tail-clipped for genotyping, ear-tagged, re-caged 4 per cage, in polysulfone cages that were not environmentally enriched (part #: RC71D-UD Udel® Polysulfone, Alternative Design, Siloam Springs, AR, 18.4 cm wide, 29.2 cm deep, 12.7 cm high, 419.35 cm^2^ floor area), with same sex, and switched from an *ad libitum* 6% fat, breeding diet (catalog #: 7002, Teklad 6% Fat Mouse/Rat Diet, Harlan, Indianapolis, IN) to an *ad libitum* 4% fat standard laboratory chow diet (catalog #: 7002, Teklad 4% Fat Mouse/Rat Diet, Harlan) and weighed daily until post-weaning day 50.

### Mouse Locomotor Activity Measurements

Locomotor activity was measured using an automated system (Med Associates, St. Albans, VT). Mice were placed singly in plastic activity chambers (12×18×33 cm) with 10 pairs of photocell beams dividing the chamber into 10 rectangular fields for measurement of total beam breaks, as described previously [7]. 8 week old mice were placed into the cages and their ambulatory locomotor activity was monitored for 2 hrs. Data are presented as number of beam brakes after 30 min and after 2 hr.

### Comparison and Histochemical Analysis of Visceral Adipose Tissue

Epididymal, mesenteric and peri-renal adipose tissue depots were excised from age-matched 2–3.5 month old male *rgs9+/+* and *rgs9-/-* mice, fixed in 10% (vol/vol) buffered formalin and then separately weighed. The mass of peri-renal adipose tissue obtained from each mouse was too small to be accurately weighed and therefore the peri-renal depot was not used in the subsequent analyses.

For histological studies of the adipose tissues, the formalin fixed tissues were dehydrated through graded ethanols, and embedded in paraffin. Sections were cut at 4 µm and stained with hematoxylin and eosin according to standard staining protocols [59].

Bright field images of hemotoxylin and eosin stained sections of mouse fat pads were captured using a 40X objective and Nikon (Melville, NY) Eclipse TS100 microscope. The adipocyte images were analyzed using the automated object/cell counting and analysis features of NIS-Elements software (Nikon). The software parameters were manually adjusted to maximize the detection and outlining of adipocytes borders in each image. Misidentified cells and partial cell images at the edges at the edges of each picture were manually excluded prior to analysis.

### Western blotting

Epididymal fat pads and striatal tissue was dissected from wild-type (*RGS9+/+*) mice and Dounce homogenized in 1X phosphate buffered saline (PBS) containing a cocktail of protease inhibitors (catalog #: S8830, Sigma-Aldrich, St. Louis, MO), solubilized in PBS containing 2% w/v sodium dodecyl sulfate (SDS) and sonicated to reduce sample viscosity. Protein concentration of resulting samples was determined using a BCA protein assay kit (catalog #: IJ116707, Pierce/Thermo Scientific, Rockford, IL). 60 µg of total protein, diluted in SDS-sample buffer containing 2% SDS, 100 mM Tris-HCl, and 50 mM dithiothreitol (DTT) was loaded per well of a 10-well mini-gel plate (mini-PROTEAN 1-dimensional electrophoresis system, Bio-Rad, Hercules, CA), resolved by SDS-polyacrylamide gel electrophoresis (PAGE), transferred to polyvinylidene fluoride (PVDF) membrane and probed using a previously validated primary polyclonal sheep antibody that specifically recognizes both long and short RGS9 isoforms [23] and an anti-sheep horse-radish peroxidase (HRP)-conjugated secondary (Jackson ImmunoResearch, West Grove, PA). A luminescent signal from the HRP-conjugated secondary was elicited using a chemiluminescent HRP substrate (catalog #: 34096, Thermo Scientific) and visualized using the Gel-Doc imaging system (Bio-Rad).

### Herpes simplex virus (HSV) constructs

The HSV vector used for overexpressing mouse RGS9-2 (Genbank accession number: AF125046) has been previously described and validated [7], [24]. The description of the control HSV-LacZ (β-galactosidase) vector is provided elsewhere [60].

After cloning, HSV constructs were grown and purified according to previously published protocols [61], [62].

### Rat Nucleus Accumbens (NAc) Protein Overexpression

All surgery was performed under anesthetic procedures described below, and all efforts were made to minimize suffering. Rat experiments were performed using one or more sets of 12 male Sprague-Dawley rats (250–275 gm, Charles River, Raleigh, NC): 6 rats were injected with Herpes simplex virus (HSV) transducing an RGS protein construct, while the remaining 6 control rats were injected with HSV transducing LacZ. Since RGS9-2 is thought to be a specific modulator of striatal D2-dopamine receptors (D2R) we attempted to balance the groups for inter-individual differences in mesostriatal dopaminergic tone, by measuring the levels of cocaine-induced locomotor activity. Cocaine administration elevates extracellular dopamine levels in the nucleus accumbens (NAc) [63], resulting in activation of striatal dopamine receptors [64] and consequent behavioral responses such as enhanced locomotion [65]. Thus the cocaine-induced locomotor response was used as a surrogate measure to balance inter-individual differences in striatal dopamine receptor function and consequently enhance our ability to detect statistically significant weight alterations produced during the short 3–4 day window of HSV-induced protein expression [7]. The balancing procedure was as follows: Following a 1 week habituation to the vivarium, rats were tested for locomotor responses to 10 mg/kg cocaine in an automated, infrared beam-based locomotor activity recording chamber and were assigned to either the experimental (HSV-RGS) or control (HSV-LacZ) groups, so that the groups were roughly balanced for the cocaine-induced locomotor activity.

The following day, rats were anesthetized (ketamine hydrochloride, 100 mg/kg and sodium pentobarbital, 30 mg/kg, i.p.) and stereotaxically injected with 2 µL of either control LacZ virus or the RGS protein transducing virus [62] bilaterally into the NAc shell using a 26 gauge Hamilton syringe, arm mounted at 10° to vertical, at A–P = +1.9, M–L = ±2.4 and D–V = −6.7 relative to bregma [66]. Expression of the HSV-delivered transgenes is detected within 24 hr post injection, reaches a maximum between days 4 and 5 and then rapidly ceases after day 6 [61], [67]. After recovery, rats continued to be fed *ad libitum* on a 4% fat rodent diet (Harlan). On days 1-5 post-HSV injection, rats were weighed between 5 and 7 hr after lights-on. At the end of the experiments, rats were perfusion-fixed and syringe tip placement in the NAc shell confirmed by histological analysis of Nissl-stained sections through the NAc. Data from animals with injection placements outside of the NAc shell were excluded from analysis (∼8% of the injected animals). The above experiment was repeated with each construct at least 3 times.

## Supporting Information

Table S1 Table describing the ethnic, sex and ΔTTTCT deletion positive (Δ+) and ΔTTTCT (Δ-) deletion negative composition of the human study sample.
(DOC)

Table S2 Detailed table describing the East Asian study population.
(DOC)
