# Supplementary material for: Association between Regulator of G Protein Signaling 9–2 and Body Weight
Source: PLoS One. 2011 Nov 23;6(11):e27984. doi: 10.1371/journal.pone.0027984 (PMC3223194; doi:10.1371/journal.pone.0027984)
Supplement: Table S1 — Table describing the ethnic, sex and ΔTTTCT deletion positive (Δ+) and ΔTTTCT (Δ-) deletion negative composition of the human study sample. (DOC) [file pone.0027984.s001.doc]

**Supporting Information Table S1.**

Table describing the ethnic, sex and ΔTTTCT deletion positive (Δ+) and ΔTTTCT (Δ-) deletion negative composition of the human study sample.

|  |  | ***No. of Subjects*** | | |
| --- | --- | --- | --- | --- |
| ***Ethnicity*** | ***Sex*** | ***Δ+*** | ***Δ-*** | ***(Δ+ & Δ-)*** |
| **Chinese** (45.0%)^a^ | **M** | 56 | 46 | 102 |
|  | **F** | 57 | 62 | 119 |
|  | **MF** | 113 | 108 | 221 |
| **Japanese** (15.9%)^a^ | **M** | 15 | 18 | 33 |
|  | **F** | 16 | 29 | 45 |
|  | **MF** | 31 | 47 | 78 |
| **Korean** (4.7%)^a^ | **M** | 2 | 9 | 11 |
|  | **F** | 2 | 10 | 12 |
|  | **MF** | 4 | 19 | 23 |
| **Pacific Islander** (21.0%)^a^ | **M** | 31 | 20 | 51 |
|  | **F** | 30 | 22 | 52 |
|  | **MF** | 61 | 42 | 103 |
| **South-East Asian** (13.4%)^a^ | **M** | 20 | 20 | 40 |
|  | **F** | 17 | 9 | 26 |
|  | **MF** | 37 | 29 | 66 |
| **All East Asians** (100%)^a^ | **M** | 124 | 113 | 237 |
|  | **F** | 122 | 132 | 254 |
|  | **MF** | 246 | 245 | 491 |

Table Key:

***Δ+***, ΔTTTCT deletion positive; ***Δ-***, ΔTTTCT deletion negative; **M**, males, **F**, females, **MF**, males and females

^a^Percentages in parenthesis in the first column indicate percent fraction of the study population that belong to each ethnic group.

|  |  | ***Δ+*** | ***Δ-*** | ***(Δ+ & Δ-)*** | ***Δ+*** | ***Δ-*** | ***(Δ+ & Δ-)*** | ***Δ+*** | ***Δ-*** | ***(Δ+ & Δ-)*** |
| --- | --- | --- | --- | --- | --- | --- | --- | --- | --- | --- |
|  |  | **Height^#^** | **Weight^§^** | ***BMI*** | **Height^#^** | **Weight^§^** | ***BMI*** | **Height^#^** | **Weight^§^** | ***BMI*** |
| **C** | **M** | 169.0±6.7 | 72.3±10.9 | ***25.2±3.1*** | 169.6±5.7 | 71.3±10.6 | ***24.7±3.0*** | 169.3±6.2 | 71.8±10.7 | 25.0±3.0 |
|  | **F** | 158.0±4.7 | 58.8±15.0 | ***23.5±5.5*** | 157.8±5.3 | 55.0±8.0 | ***22.1±3.4*** | 157.9±5.0 | 56.8±12.0 | 22.8±4.6 |
|  | **MF** | 163.5±8.0 | 65.5±14.7 | ***24.4±4.6*** | 162.8±8.0 | 61.9±12.3 | ***23.2±3.5*** | 163.1±8.0 | 63.7±13.6 | 23.8±4.1 |
| **J** | **M** | 167.0±10.6 | 75.4±20.0 | ***26.8±6.1*** | 170.0±7.6 | 73.0±13.2 | ***25.3±4.4*** | 168.6±9.1 | 74.1±16.4 | 26.0±5.2 |
|  | **F** | 156.5±5.1 | 57.5±13.4 | ***23.4±4.8*** | 156.6±4.7 | 58.4±11.0 | ***23.8±4.0*** | 156.5±4.8 | 58.1±11.8 | 23.6±4.2 |
|  | **MF** | 161.5±9.7 | 66.2±19.0 | ***25.1±5.6*** | 161.7±8.8 | 64.0±13.8 | ***24.4±4.2*** | 161.6±9.1 | 64.9±16.0 | 24.6±4.8 |
| **K** | **M** | 163.8±5.4 | 72.4±2.9 | ***27.0±2.9*** | 166.5±6.6 | 72.6±10.0 | ***26.1±2.8*** | 166.0±6.2 | 72.6±9.0 | 26.3±2.7 |
|  | **F** | 160.0±7.2 | 55.6±8.0 | ***21.6±1.2*** | 151.2±7.8 | 54.5±11.3 | ***21.8±4.1*** | 158.4±7.4 | 54.7±10.5 | 21.7±3.7 |
|  | **MF** | 161.9±5.6 | 64.0±10.9 | ***24.3±3.6*** | 162.1±8.3 | 63.1±14.0 | ***23.8±4.1*** | 162.1±7.8 | 63.2±13.3 | 23.9±4.0 |
| **PI** | **M** | 165.1±8.0 | 73.8±10.9 | ***26.9±3.3*** | 170.1±8.1 | 79.8±11.9 | ***27.6±3.5*** | 167.3±8.2 | 76.1±11.6 | 27.2±3.4 |
|  | **F** | 159.9±7.3 | 64.1±14.2 | ***24.9±4.2*** | 156.6±8.0 | 59.6±14.7 | ***24.1±4.2*** | 158.5±7.7 | 62.2±14.4 | 24.5±4.2 |
|  | **MF** | 162.7±8.1 | 69.0±13.4 | ***25.9±3.9*** | 163.0±10.5 | 69.2±16.8 | ***25.7±4.2*** | 162.8±9.1 | 69.1±14.8 | 25.8±4.0 |
| **SEA** | **M** | 167.1±7.9 | 72.2±10.7 | ***25.8±3.1*** | 168.5±6.1 | 73.4±12.7 | ***25.8±4.2*** | 167.8±7.0 | 72.8±11.6 | 25.8±3.6 |
|  | **F** | 157.9±6.8 | 60.5±17.4 | ***24.2±4.8*** | 156.6±4.6 | 59.6±15.7 | ***24.3±6.7*** | 157.5±4.6 | 60.2±12.0 | 24.2±4.7 |
|  | **MF** | 162.9±8.0 | 66.8±11.8 | ***25.1±3.3*** | 164.8±8.0 | 69.1±14.9 | ***25.3±5.0*** | 163.8±8.0 | 67.8±13.2 | 25.2±4.1 |
| **AEA** ( | **M** | 167.5±7.8 | 73.0±12.1 | ***26.0±3.7*** | 169.3±6.6 | 73.6±11.8 | ***25.6±3.6*** | 168.4±7.3 | 73.3±11.9 | 25.8±3.6 |
|  | **F** | 158.3±5.6 | 60.1±13.9 | ***23.9±4.8*** | 157.3±5.8 | 56.8±10.9 | ***22.9±4.0*** | 157.8±5.7 | 58.4±12.5 | 23.4±4.5 |
|  | **MF** | 162.9±8.2 | 66.6±14.5 | ***24.9±4.4*** | 162.8±8.6 | 64.5±14.1 | ***24.2±4.1*** | 162.9±8.4 | 65.6±14.3 | 24.6±4.2 |

**Supporting Information Table S2**

Detailed table describing the East Asian study population

Table Key:

***Δ+***, ΔTTTCT deletion positive; ***Δ-***, ΔTTTCT deletion negative; **C**, Chinese; **J**, Japanese; **K**, Koreans; **PI**, Pacific Islanders; **SE**, South-East Asians; **AEA**, All East Asians, **M**, males, **F**, females, **MF**, males and females.

^#^Height is denoted in cm; ^§^Weight is denoted in kg.
